# MK5 Regulates Microglial Activation and Neuroinflammation in Experimental Stroke Models

**DOI:** 10.1111/cns.70395

**Published:** 2025-04-16

**Authors:** Xingzhi Wang, Wenqi Mao, Li Du, Fei Wang, Ye Pang, Yangdanyu Li, Guangci Xu, Guiyun Cui

**Affiliations:** ^1^ Department of Neurology The Affiliated Hospital of Xuzhou Medical University Xuzhou China; ^2^ Institute of Stroke Research Xuzhou Medical University Xuzhou China; ^3^ Department of Neurology Xuzhou New Healthy Geriatric Hospital Xuzhou Jiangsu China; ^4^ Department of Neurology The First People's Hospital of Sihong Count Suqian Jiangsu China; ^5^ Department of Neurology Xuzhou Medical University Affiliated Hospital Sihong Branch Suqian Jiangsu China

**Keywords:** HSP27, ischemic stroke, microglia, MK5, neuroinflammation

## Abstract

**Objective:**

Microglial activation plays a crucial role in neuroinflammation following ischemic stroke. This study was conducted to investigate the role and potential mechanisms of MK5 within microglial cells in the inflammatory response following ischemic stroke in mice in vivo and in vitro.

**Methods:**

Microglia‐specific conditional MK5 knockout (MK5 cKO) mice and their control mice (MK5^f/f^) were subjected to middle cerebral artery occlusion (MCAO). BV2 cells (a mouse microglial cell line) were transfected with small interfering RNA (siRNA) to knock down MK5 levels and subsequently exposed to oxygen–glucose deprivation/reperfusion (OGD/R) to simulate ischemic conditions in vitro. Following MCAO, behavioral tests and infarct volume measurements were conducted. Levels of cytokines and microglial markers were evaluated using qPCR and Western blotting, while immunofluorescence was employed to observe microglial activation. Additionally, Western blotting was performed to assess the phosphorylation of HSP27 and NF‐κB.

**Results:**

Compared to the control group, the knockout of the MK5 gene in microglia significantly exacerbated neurological deficits and increased infarct volume in MCAO mice. The loss of the MK5 promoted inflammation by upregulating pro‐inflammatory factors and downregulating anti‐inflammatory factors, while also enhancing microglial activation in both MCAO mice and BV2 microglial cells subjected to OGD/R. Furthermore, the knockout of the MK5 gene in microglia reduced the phosphorylation levels of HSP27 and increased the phosphorylation levels of NF‐κB in the aforementioned models.

**Conclusion:**

Microglial MK5 plays a critical role in the ischemic neuroinflammatory response by regulating the phosphorylation of HSP27 and NF‐κB, positioning it as a potential target for stroke treatment.

## Graphical abstract

MK5 gene knockout promotes microglial activation by regulating the phosphorylation of HSP27 and NF‐κB, thereby exacerbating functional decline.

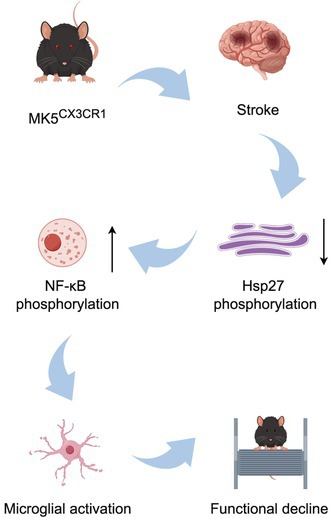

## 1 Introduction

Stroke is a leading cause of disability and the second most common cause of death worldwide [1]. The most prevalent type, ischemic stroke, accounts for 75%–90% of all cases, and its incidence is rising due to an aging population [2]. Despite advancements in intravenous and mechanical thrombolysis, which have somewhat improved recovery in ischemic stroke patients, treatment options remain extremely limited due to the narrow “time window” for these therapies [3]. Therefore, there is an urgent need to explore alternative therapeutic strategies.

Neuroinflammation, primarily driven by the excessive activation of resident microglia, plays a critical role in the pathogenesis of ischemic stroke. Although microglia contribute to mitigating brain injury during the recovery phase, their inflammatory responses often overshadow their protective effects during the acute phase of ischemic stroke [4]. During the acute phase of ischemic stroke, activated microglia initially produce anti‐inflammatory factors to protect the brain. However, after approximately 3 days to 1 week, pro‐inflammatory microglia increase, releasing mediators such as high mobility group box 1 (HMGB1), matrix metalloproteinases (MMPs), and interleukin‐1β (IL‐1β), which promote immune cell infiltration and ultimately compromise the blood–brain barrier integrity [5]. These cells enhance the expression of adhesion molecules on leukocytes and endothelial cells by releasing mediators such as high mobility group box 1 (HMGB1), matrix metalloproteinases (MMPs), and interleukin‐1β (IL‐1β). This release facilitates the infiltration of neutrophils, blood‐derived macrophages, and T‐cells into the ischemic region, which ultimately compromises the integrity of the blood–brain barrier [6]. Thus, investigating the underlying pathological mechanisms and identifying neuroprotective agents that can specifically target and suppress microglia‐mediated neuroinflammation may facilitate the development of novel therapeutic strategies for ischemic stroke.

MK5 is a protein kinase activated by mitogen‐activated protein kinase kinase 5 (MAPKK5), and it is widely expressed in human and murine tissues and cell lines, with particularly high expression in the brain, heart, liver, skeletal muscle, and kidney [7, 8, 9]. As a homolog of MK2 and MK3, MK5 is part of the family of protein kinases activated by p38/MAPK and is also known as p38‐regulated/activated protein kinase (PRAK) [10]. Previous studies have established that the p38/MAPK pathway is a prototypical inflammatory signaling pathway. Altered phosphorylation levels of p38 MAPK during stroke have been shown to regulate neuronal survival and apoptosis [11, 12]. Recent studies have demonstrated that the p38/MAPK signaling pathway mediates pro‐inflammatory responses in microglia during ischemic stroke [13, 14]. However, as a downstream target of the p38/MAPK pathway, it remains uncertain whether MK5 is involved in immune inflammatory responses during ischemic stroke, especially in microglia‐mediated neuroinflammation. Therefore, this study employed Microglia‐specific conditional MK5 knockout (MK5 cKO) mice to knockout microglial MK5 in the brain, followed by middle cerebral artery occlusion (MCAO), while BV2 cells were transfected with small interfering RNA (siRNA) to knock down MK5 levels, and subsequently exposed to oxygen–glucose deprivation/reperfusion (OGD/R) to investigate the role of microglial MK5 in microglial activation and ischemic stroke progression. The objective was to establish a theoretical basis for targeting MK5 as a potential therapeutic intervention for ischemic stroke.

## 2 Materials and Methods

### 2.1 Animals

C57BL/6J WT mice with specified pathogen‐free (SPF) status were purchased from Shandong Jiyuan Experimental Animal Breeding Co. Ltd., Jinan, and approved by the Medical Ethics Committee of Xuzhou Medical University. SPF‐grade MK5 conditional gene knockout mice (MK5^f/f^ mice) were purchased from EMMA (European Mouse Mutant Archive, Germany), and CX3CR1‐CreER mice were purchased from Shanghai Model Organisms Center Ltd. To generate microglia‐specific MK5 conditional knockout mice, CX3CR1‐Cre mice were crossed with MK5^f/f^ mice [15, 16]. In this study, adult male MK5^f/f^; CX3CR1‐Cre mice, in which MK5 is specifically deleted in microglia, along with their wild‐type littermates (MK5^f/f^ mice), were used. Genotyping was performed at 10 days of age using tail samples and PCR analysis. At 8 weeks of age, mice were intraperitoneally injected with tamoxifen (100 mg/kg, Sigma‐Aldrich, USA) on days 1, 3, and 5 to achieve conditional MK5 gene knockout.

All mice were allowed access to food and water ad libitum and maintained at 22°C –24°C with a 12 h light/dark cycle. All animal experiments were carried out in compliance with the ethical regulations and approved by the Institutional Animal Care and Use Committee of the Xuzhou Medical University Experimental Animal Department.

### 2.2 Genotyping

The genotype of mice was determined via PCR using genomic DNA (gDNA) extracted from their toes. Briefly, a 2‐mm section of the tail was digested in 75 μL of 25 mM NaOH containing 0.2 mM EDTA at 100°C for 1 h. Subsequently, 75 μL of 40 mM Tris–HCl (pH 5.5) was added, and the samples were centrifuged at 5000 rpm for 2 min. The supernatant containing gDNA was collected for subsequent genotyping. The PCR system consisted of the following components: 10 μL of 2× Taq Plus Master Mix (Vazyme, P212), 0.5 μL of forward primer (10 pmol/μL); 0.5 μL of reverse primer (10 pmol/μL), and 9 μL of gDNA. The cycling reaction was step 1, 94°C for 3 min; step 2, 35 cycles of 94°C for 30 s, 60°C for 30 s, and 72°C for 30 s; step 3, 72°C for 5 min; and step 4, holding at 12°C. The primers used for genotyping were as follows: MK5 loxp: forward (5′‐GACCCATCTGAGTGAGCCA‐3′) and reverse (5′‐GGTGTTTGGAGTAAAAAGGAGG‐3′); CX3CR1‐Cre: forward (5′‐GCCTGCATTACCGGTCGATGCAACGA‐3′) and reverse (5′‐GTGGCAGATGGCGCGGCAACACCATT‐3′).

### 2.3 Middle Cerebral Artery Occlusion (MCAO) Model

The MCAO mouse model was established to simulate stroke following the protocol outlined in our previous study [17]. Briefly, mice were initially anesthetized with 3% isoflurane in oxygen, and anesthesia was maintained by adjusting the concentration to 1.5% as required. Following a midline incision in the neck, the proximal common carotid artery and the external carotid artery were ligated. A silicone rubber‐coated 6.0 nylon monofilament (Doccol, Redland, CA, USA) was then introduced through the right internal carotid artery and advanced to occlude the origin of the middle cerebral artery. After 60 min of occlusion, reperfusion was achieved by removing the filament. Sham‐operated mice underwent identical anesthesia and surgical exposure but did not receive MCAO induction.

### 2.4 Behavioral Tests

Before performing the MCAO procedure, all mice underwent a 5‐day training regimen with bi‐daily sessions to reduce anxiety and establish consistent baseline conditions across control and experimental groups. Neurological function was assessed using the modified neurological severity score (mNSS), corner test, cylinder test, rotarod test, and foot fault test, both prior to surgery and at 3 days post‐surgery.

The mNSS comprises multiple assessments, including motor function tests, the timed beam balance test, sensory tests (visual and tactile), reflex responses to sudden auditory stimuli, and the corneal reflex test. Neurological function is rated on a scale from 0 to 14, where a score of 0 indicates normal function and a score of 14 represents the maximum deficit [18]. The corner test was performed as previously outlined [19]. Mice were placed in a 30° angle corner formed by two cardboard pieces, each measuring 30 × 20 × 1 cm^3^. Upon entering the deep part of the corner, the mice would rear up and then turn to exit the corner. They could turn either left or right, and the direction of the turn was recorded. Each mouse underwent 20 trials, and the percentage of right turns was calculated. The cylinder test evaluates locomotor asymmetry [19]. A mouse is placed in a transparent cylinder (9 cm in diameter, 15 cm in height) and its forelimb movements are observed as it makes contact with the cylinder wall. Each contact is recorded based on whether the forelimb is impaired or nonimpaired. Simultaneous contact of both forelimbs is noted as a bilateral movement. If the impaired forelimb touches the wall first and the nonimpaired forelimb touches afterward while the impaired forelimb remains in contact, both movements are recorded. Lateral exploration with alternating forelimbs is recorded as two movements. A total of 20 movements is recorded over 10 min. The final score is calculated as: (nonimpaired forelimb movements – impaired forelimb movements)/(nonimpaired forelimb movements + impaired forelimb movements + bilateral movements). The foot fault test assessed sensorimotor deficits by placing individual mice on a 10‐mm square wire mesh elevated 40 cm × 40 cm. Mice were allowed to walk freely for 2 min while being videotaped. The number of foot faults and total steps were recorded, and the percentage of foot faults was calculated as the ratio of foot faults to total steps [20]. As detailed in our previous studies, the Rotarod test involved placing experimental mice in a rotating drum (ZH‐600B; Zhenghua Bio) that ramped up from 5 to 40 rpm over a 5‐min period. Each mouse underwent three consecutive tests over 3 days before surgery and was tested again on the third day post‐surgery, with a 15‐min interval between each test [21].

### 2.5 TTC Staining

After anesthetizing mice with 1% pentobarbital sodium, brains were quickly extracted 3 days post‐MCAO. The fresh brains were stored at −20°C for 30 min before being sectioned into 2 mm coronal slices. These slices were then immersed in a 2% TTC solution (Sigma‐Aldrich, USA) in phosphate buffer and stained in the dark at 37°C for 20 min. The sections were fixed overnight at 4°C in a 4% paraformaldehyde (PFA) solution. TTC‐stained sections were imaged using a digital camera, and infarct areas were quantified with ImageJ software. The total infarct volume was calculated using the formula: (contralateral hemisphere volume – non‐infarcted ipsilateral hemisphere volume)/contralateral hemisphere volume × 100%.

### 2.6 Cerebral Blood Flow (CBF) Measurements

The laser speckle imaging system (RWD, Shenzhen, China) was used to measure cortical blood flow according to the manufacturer's protocol. Briefly, a charge‐coupled device (CCD) image sensor was positioned above the anesthetized mouse's head, and a 785 nm laser diode illuminated the skull to enable diffuse laser penetration through the brain. Cerebral blood flow (CBF) was assessed bilaterally and recorded 15 min before MCAO induction, throughout ischemia, and up to 15 min post‐reperfusion. Mice were shielded from direct sunlight and infrared radiation, with room temperature maintained at 26°C. To evaluate CBF changes, a region of interest (ROI) was defined to include the right cortical infarct area, posterior to the coronal suture and medial to the linear temporalis. Mice with a CBF reduction of less than 75% compared to baseline or a mortality rate below 10% were excluded, as early CBF drops below 25% of control indicated a high probability (> 95%) of infarction [22].

### 2.7 Cell Culture, siRNA Transfection, and OGD/R Treatment

Building upon and improving the methods established by Milanova et al. [23], primary microglial cells were isolated from the brains of neonatal MK5^f/f^ mice and MK5^f/f^; CX3CR1‐Cre mice. Brain tissue was dissected to remove meninges and blood vessels, followed by enzymatic digestion with trypsin–EDTA. Dissociated cells were plated onto poly‐L‐lysine‐coated flasks with Dulbecco's modified Eagle's medium (DMEM, Gibco, USA) containing 10% fetal bovine serum (FBS, Gibco, USA) and 1% penicillin–streptomycin (Gibco, USA). After 7 days, 0.25 ng/mL of granulocyte‐macrophage colony‐stimulating factor (CSF2/GM‐CSF, Peprotech, Rocky Hill, NJ, USA) was added to promote proliferation. Microglia were detached by gentle shaking and collected through centrifugation at 1500 *g* for 5 min to evaluate knockout efficiency. All cells were treated with culture medium containing 1 μM 4‐hydroxytamoxifen (Sigma‐Aldrich, St. Louis, MO) for 48 h before assessing MK5 protein levels.

BV2 cells (catalog number SCSP‐5208) were originally purchased from the Cell Bank of Type Culture Collection of the Chinese Academy of Sciences (Shanghai, China) and have since been preserved in our laboratory. The cells were cultured in 6‐well plates with DMEM containing high glucose, supplemented with 10% FBS, 120 U/mL penicillin, and 100 mg/L streptomycin. The cultures were incubated at 37°C in an incubator with 5% CO_2_.

Small interfering RNA (siRNA) directed against mouse MK5 was utilized to achieve the silencing of MK5 expression. A mixture of siRNA (5 pmol, catalog no. 4390771; ID: s69586; Thermo Fisher Scientific) and Lipofectamine 2000 (2 μL, Invitrogen) was incubated at room temperature for 15 min. This mixture was then added to microglial cells and allowed to incubate for 24 h.

Following 36 h of siRNA transfection, BV2 microglial cells were subjected to 3 h of in vitro oxygen–glucose deprivation (OGD) to mimic ischemic conditions. Briefly, the culture medium was replaced with glucose‐free medium, and the cells were placed in a humidified incubator at 37°C under a gas mixture of 1% O_2_, 5% CO_2_, and 94% N_2_ for 3 h. After this, the cells were returned to normal culture medium and incubated at 37°C with 5% CO_2_ for an additional 24 h to allow for reperfusion.

### 2.8 Western Blotting

Total proteins were extracted from ischemic penumbra tissue or BV2 cells subjected to OGD/R using RIPA lysis buffer (P0013B; Beyotime, China), supplemented with phosphatase and protease inhibitors in appropriate proportions. An enhanced BCA Protein Assay Kit (P0010; Beyotime, China) was then used to measure the protein concentration. The samples were boiled at 100°C and separated by SDS–PAGE. The proteins were transferred onto nitrocellulose (NC) membranes (Millpore, USA). NC membranes were blocked with 5% skim milk at room temperature for 2 h, incubated with primary antibodies overnight at 4°C, and then incubated with secondary antibodies bound to HRP at room temperature for 2 h. The relative abundances of the target proteins were detected using Super ECL detection reagent (36208ES76; YEASEN, Shanghai, China) and analyzed with ChemiDocTMXRS+ and Image LabTM Software (721BR13813; Bio‐Rad, USA). ImageJ software (NIH, Bethesda, Maryland, USA) was used to analyze the gray values of each band. GAPDH was used as an internal standard for statistical analysis. The primary antibodies were as follows: MK5 (1:1000, mouse; sc‐46667, Santa Cruz, USA), Iba‐1 (1:1000, rabbit; 17198, CST, USA), IκB (1:1000, rabbit; 4812, CST, USA), phospho‐IκB (1:1000, rabbit; 2859, CST, USA), HSP27 (1:1000, rabbit; 2442, CST, USA), phospho‐ HSP27 (1:1000, rabbit; 9709, CST, USA), Arginase‐1 (1:1000, rabbit; 89872, CST, USA), CD206 (1:1000, rabbit; 24595, CST, USA), IL‐1β (1:1000, rabbit; 12426, CST, USA), TNF‐α (1:1000, rabbit; 3707, CST, USA), NF‐κB (1:1000, rabbit; 8242, CST, USA), phospho‐NF‐κB (1:1000, rabbit; 3033, CST, USA), and GAPDH (1:1000, mouse; 60004‐1‐Ig, Protein Tech, Wuhan, China). The secondary antibodies were as follows: HRP‐conjugated Goat Anti‐Rabbit IgG (1:2000, SA00001‐2; Protein Tech, Wuhan, China), and HRP‐conjugated Goat Anti‐Mouse IgG (1:2000, SA00001‐1; Protein Tech, Wuhan, China).

### 2.9 Immunofluorescence

Brain cryosections or treated BV2 cells were first fixed in 4% paraformaldehyde (PFA) for 20 min. Permeabilization was then carried out using a solution of 0.5% Triton X‐100 (Sigma‐Aldrich, USA), 10% donkey serum (Sigma‐Aldrich, USA), and 90% PBS for 60 min at room temperature. The samples were incubated overnight at 4°C with primary antibodies against CD16 (1:500, goat; PA5‐47230, Thermo Fisher Scientific, USA), CD68 (1:500, Rat; 14‐0681‐82, Thermo Fisher Scientific, USA), and Iba‐1 (1:500, rabbit; 019‐19741, WAKO, Japan). Following this, sections were incubated for 1 h with Alexa 594‐ or Alexa 488‐conjugated secondary antibodies (1:500, Vector Laboratories, USA), washed with PBS, and counterstained with a DAPI‐based anti‐fluorescence quenching agent. The samples were then mounted and examined using confocal microscopy (Leica STELLARIS 5, Germany). Positive cell counts were performed blindly by researchers, and all data were analyzed using ImageJ software (NIH, Bethesda, MD, USA).

### 2.10 Quantitative RT‐PCR

According to our previous research [17], total RNA was extracted from the peri‐infarct cortex of each group using Trizol (Invitrogen). After quantification, mRNA was reverse transcribed into cDNA with the HiScript Q RT SuperMix for qPCR Kit (Vazyme, China). qPCR was conducted on the Bio‐Rad CFX96 Real‐Time System with the UltraSYBR Mixture (CWBIO, China), following the manufacturer's recommended cycling conditions. Each qPCR assay was performed in triplicate. Gene expression was quantified using the $2^{-\Delta \Delta C_{T}}$ method and normalized to β‐actin. The primers used are listed in Table S1.

### 2.11 Statistical Analysis

Statistical analyses were performed using GraphPad Prism 8.0 (GraphPad Software, USA) or SPSS 26.0 (IBM Corporation, USA). Data is presented as mean ± standard deviation (SD). The Shapiro–Wilk test was used to assess normality for all data. Comparisons between two groups were conducted using the Student's *t*‐test for normally distributed variables and the Mann–Whitney *U* test for non‐normally distributed variables. For comparisons across multiple aging groups, one‐way ANOVA was applied for normally distributed data, and the Kruskal–Wallis test was used for non‐normally distributed data. Statistical significance was set at *p* < 0.05.

## 3 Results

### 3.1 Assessment of Acquisition and Growth Development in MK5 Microglia‐Specific Knockout Mice

**FIGURE 1:**
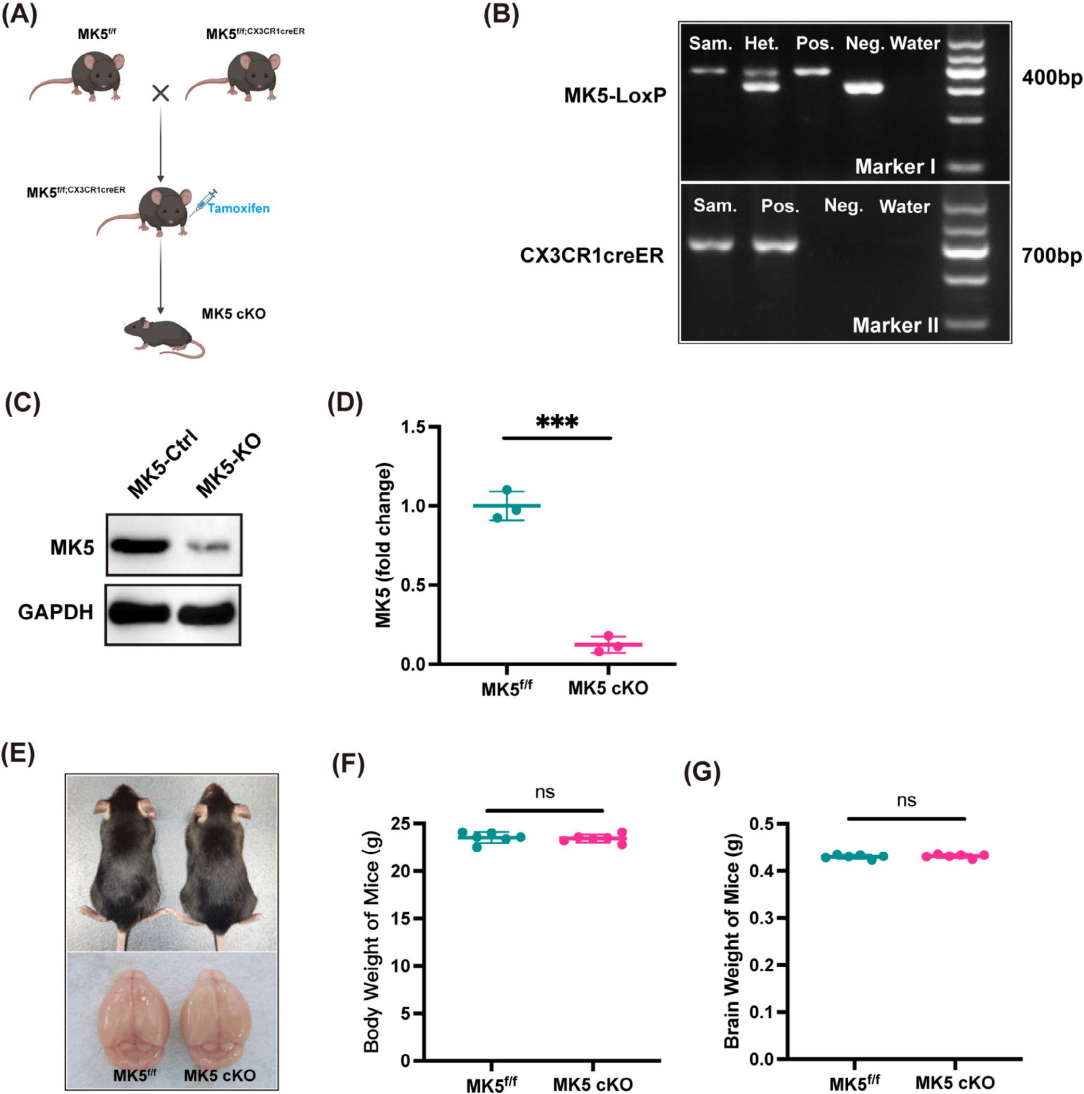
Acquisition and growth development assessment of MK5 cKO mice. (A) Diagram for breeding of homozygous MK5^f/f^ mice and MK5^f/f; CX3CR1Cre‐ER^ mice. (B) Identification of mice carrying the MK5 f/f allele and CX3CR1Cre‐ER allele, resulting in homozygous male gene mice. (C) Western blot and (D) quantification of MK5 levels in primary microglia isolated from MK5^f/f^ and MK5 cKO mice after 4‐hydroxytamoxifen treatment. *n* = 3/group, ***p < 0.001. (E–G) Comparison of body and brain weights between healthy 8‐week‐old male MK5^f/f^ and MK5 cKO mice shows no significant differences in developmental status between the two groups. *n* = 6/group. Ns, not significant.

To investigate the role and mechanism of MK5 in the inflammatory response of microglia following ischemic stroke, MK5‐LoxP mice were crossed with CX3CR1Cre‐ER mice using the Cre‐LoxP system. After several generations, MK5^f/f; CX3CR1Cre‐ER^ mice were obtained. At 8 weeks of age, tamoxifen (100 mg/kg) was administered via intraperitoneal injection every other day for a total of three doses to induce the targeted genetic modification, resulting in the desired microglia‐specific knockout of MK5 (MK5 cKO mice, Figure 1A). PCR analysis was performed to confirm the genotypes of MK5‐LoxP and CX3CR1Cre‐ER mice and to obtain homozygous male mice (Figure 1B). We subsequently isolated primary microglial cells from MK5 conditional knockout (cKO) mice to generate MK5 knockout microglia (MK5‐KO) in vitro. Primary microglia derived from MK5 ^f/f^ mice served as controls (MK5‐Ctrl). All primary microglial cells were treated with 4‐hydroxytamoxifen to facilitate MK5 gene deletion. We observed a significant reduction in MK5 protein levels in MK5‐KO microglia compared to MK5‐Ctrl (Figure 1C,D). Additionally, the growth and development status of healthy male 8‐week‐old MK5^f/f^ and MK5 cKO mice were evaluated by measuring body weight and brain weight, revealing no significant differences between the two groups (Figure 1E–G). These findings suggest that there are no discernible differences in growth and development between the two strains of mice.

### 3.2 Knockout of the MK5 Gene in Microglia Exacerbates Functional Impairments in Stroke Mice

**FIGURE 2:**
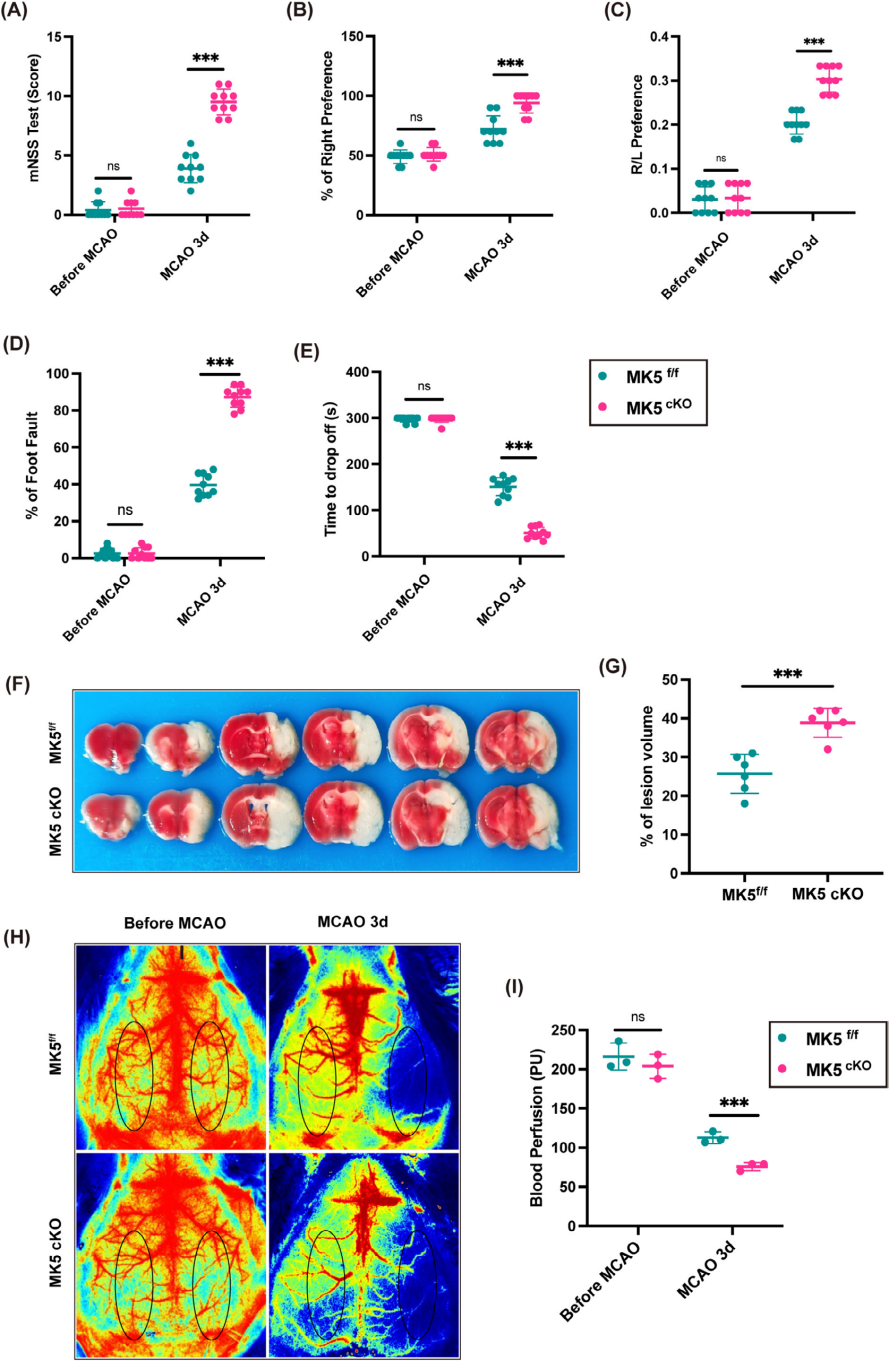
Knockout of the MK5 gene in microglia exacerbated functional impairments in MCAO mice. Neurological deficits were evaluated using the mNSS test (A), corner test (B), cylinder test (C), foot fault test (D), and rotarod test (E) before MCAO (baseline) and 3 days after MCAO. *n* = 10/group, ***p < 0.001. (F, G) Brain tissue infarct volume comparison between MK5^f/f^ and MK5 cKO mice 3 days after MCAO. *n* = 6/group, ***p < 0.001. (H, I) Peri‐infarct blood flow changes and quantitative cerebral blood flow perfusion in MK5^f/f^ and MK5 cKO mice before and 3 days after MCAO. *n* = 3/group, ***p < 0.001.

To investigate the role of MK5 in microglial cells following ischemic stroke, sensory‐motor functions were assessed in two groups of mice before stroke and on the third day post‐stroke. Compared to the control group, microglia‐specific conditional MK5 knockout mice exhibited significantly worsened neurological deficit scores, increased turn counts in the corner test, higher forelimb asymmetry scores in the cylinder task, more foot faults in the grid‐walk task, and impaired motor performance in the rotarod task (Figure 2A–E). These results indicate exacerbated sensory‐motor impairments. Additionally, TTC staining to measure infarct volume in brain tissue revealed a significant increase in infarct volume in MK5 knockout mice 3 days after stroke (Figure 2F,G). Laser speckle flow imaging also demonstrated that microglia‐specific conditional MK5 knockout significantly reduced perfusion in the ischemic core and surrounding brain tissue on the third day post‐stroke (Figure 2H,I).

### 3.3 Knockout of the MK5 Gene in Microglia Enhances Inflammatory Activation Following Stroke

**FIGURE 3:**
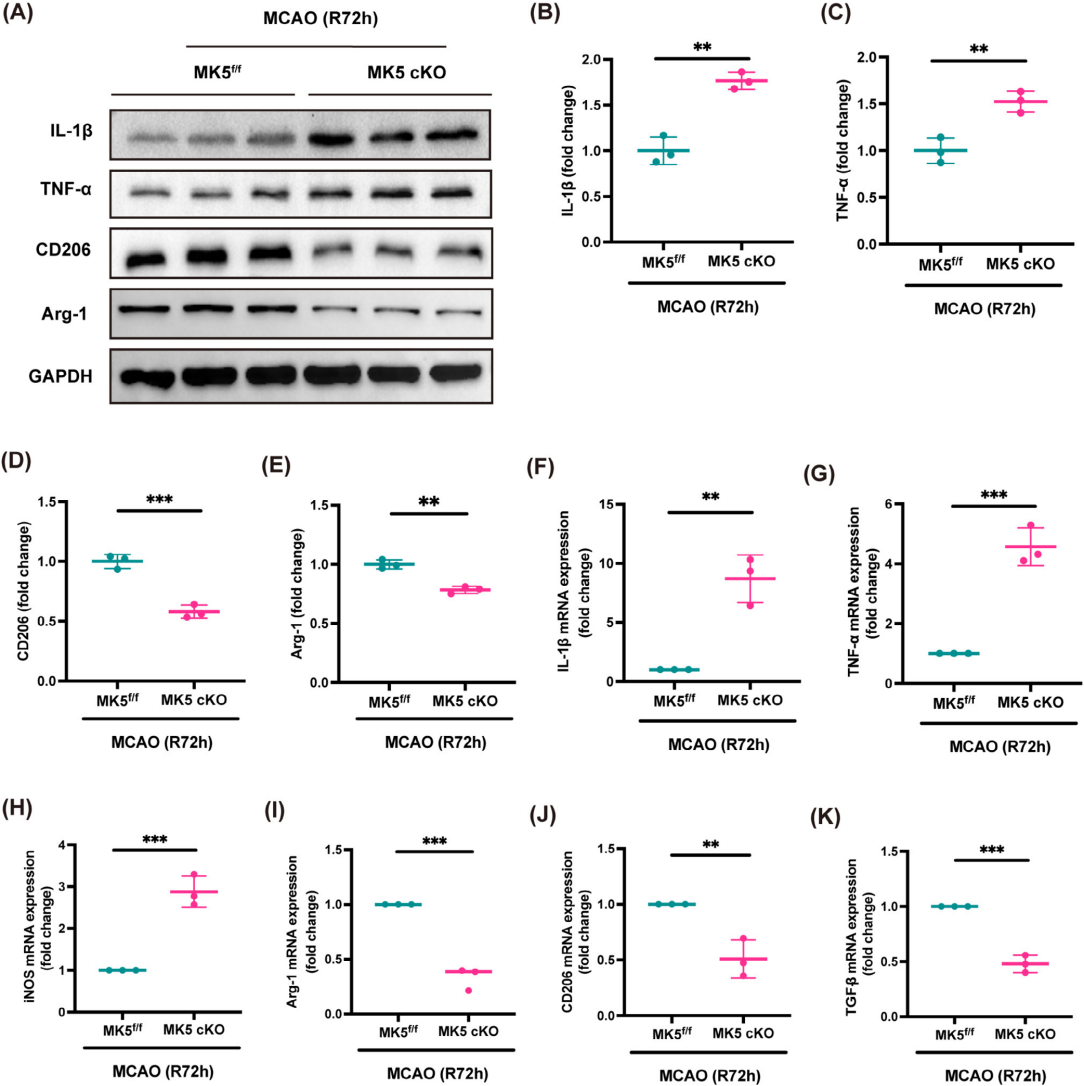
Impact of MK5 deletion in microglia on inflammatory cytokines protein and mRNA expression in MCAO mice. (A–E) Western blot analysis of pro‐inflammatory cytokines IL‐1β and TNF‐α, and anti‐inflammatory markers CD206 and Arg‐1 in the peri‐infarct cortex of MK5^f/f^ and MK5 cKO mice 3 days post‐MCAO. *n* = 3/group, **p < 0.01, ***p < 0.001. (F–H) qPCR analysis of mRNA expression of pro‐inflammatory cytokines IL‐1β, TNF‐α, iNOS in the peri‐infarct cortex of MK5^f/f^, and MK5 cKO mice 3 days post‐stroke. *n* = 3/group, **p < 0.01, ***p < 0.001. (I–K) qPCR analysis of mRNA expression of anti‐inflammatory markers Arg‐1, CD206, TGF‐β in the peri‐infarct cortex of MK5^f/f^ and MK5 cKO mice 3 days post‐stroke. *n* = 3/group, **p < 0.01, ***p < 0.001.

Due to the regulatory role of MK5 in post‐stroke functional outcomes within microglia, we aimed to explore the underlying mechanisms. On the third day post‐MCAO surgery, we performed rapid dissection to collect tissue surrounding the infarct core for Western blotting and qPCR analysis. Compared to the control group, Western blotting revealed that MK5 knockout in microglia significantly increased the expression levels of pro‐inflammatory cytokines IL‐1β and TNF‐α, while simultaneously decreasing the expression of anti‐inflammatory factors CD206 and Arg‐1 (Figure 3A–E). This suggests that MK5 knockout may promote the activation of inflammatory cells following stroke. qPCR further confirmed that MK5 gene knockout affected the expression of inflammatory cytokines at the gene level, showing significant upregulation of TNF‐α, IL‐1β, and iNOS mRNA levels, and significant downregulation of Arg‐1, CD206, and TGF‐β mRNA levels (Figure 3F–K). These findings indicate that MK5 gene knockout in microglia exacerbates neuroinflammation in the peri‐infarct cortex following stroke.

### 3.4 Knockout of the MK5 Gene in Microglia Enhances Microglial Activation Following Stroke

**FIGURE 4:**
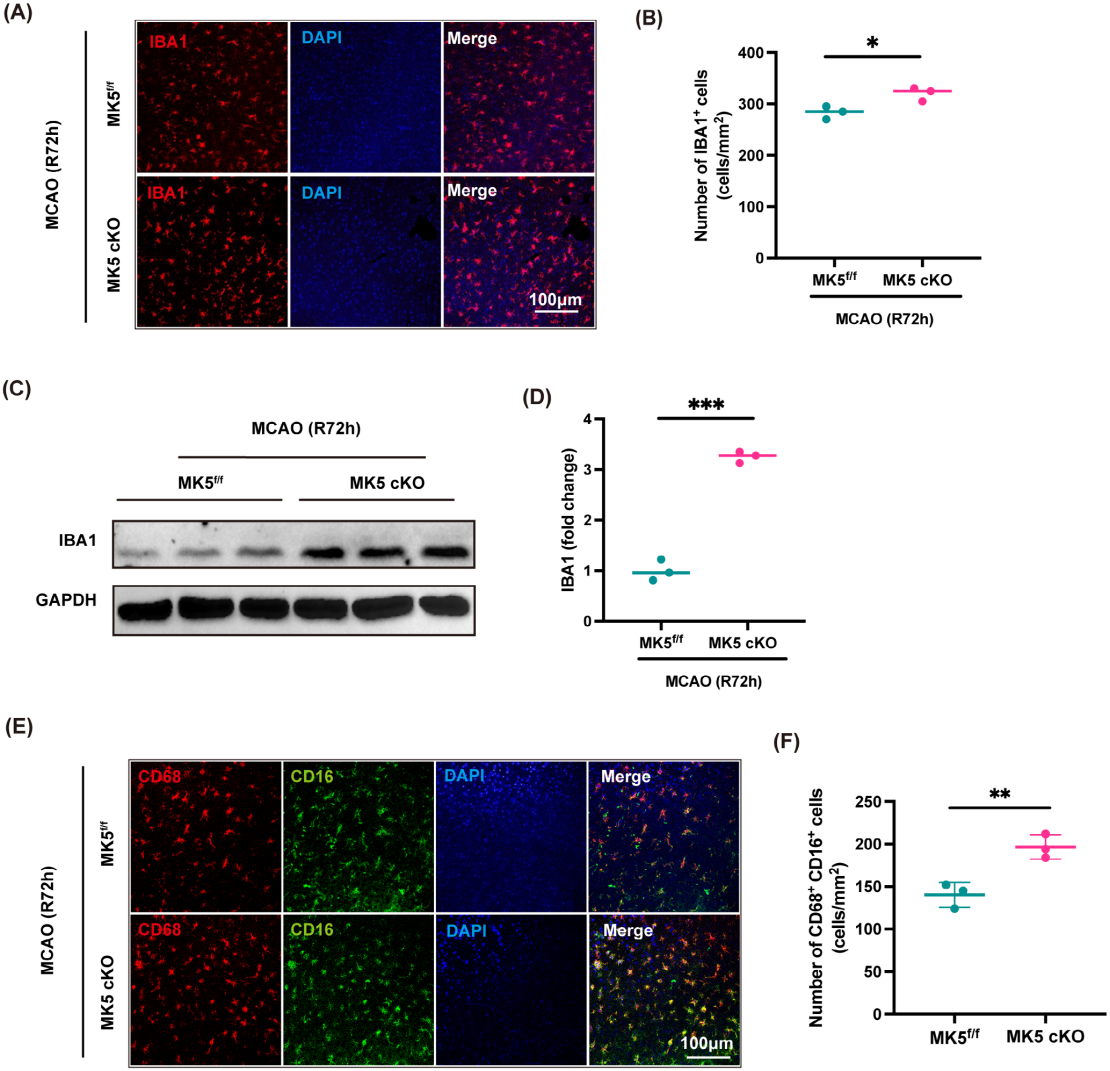
MK5 gene knockout in microglia promotes microglial activation in vivo. (A, B) Immunostaining and statistical analysis of IBA1 in the peri‐infarct cortex of MK5^f/f^ and MK5 cKO mice 3 days post‐MCAO. *n* = 3/group, *p < 0.05. Scale bar: 100 μm. (C, D) Western blot analysis and statistical analysis of IBA1 expression in the peri‐infarct cortex of MK5^f/f^ and MK5 cKO mice 3 days post‐stroke. *n* = 3/group, ***p < 0.001. (E) Immunostaining for co‐localization of CD68 and CD16 in the peri‐infarct cortex of MK5^f/f^ and MK5 cKO mice 3 days post‐stroke. Scale bar: 100 μm. (F) Statistical analysis of CD68+/ CD16+ cells in the peri‐infarct cortex of MK5^f/f^ and MK5 cKO mice. *n* = 3/group, **p < 0.01.

IBA1, a calcium‐binding protein specifically expressed in microglial cells, is widely used as a marker for these cells. Immunostaining revealed an increase in the number of IBA1^+^ cells in the peri‐infarct cortex following MK5 gene knockout in microglia, compared to the control group (Figure 4A,B). Additionally, Western blotting showed elevated levels of IBA1 protein in the ischemic penumbra tissue following MK5 gene knockout in microglia (Figure 4C,D). Furthermore, we examined the influence of MK5 gene knockout on microglial cell polarization. Immunofluorescence demonstrated a notable increase in CD68+/CD16+ cells in the peri‐infarct tissue after MK5 gene knockout in microglia compared to controls (Figure 4E,F). These results collectively indicate that knockout of MK5 in microglial cells activates inflammatory responses, driving microglial polarization towards the M1 pro‐inflammatory phenotype and thereby promoting neuroinflammation.

### 3.5 MK5 Knockdown Enhances Inflammatory Activation and Microglial Activation After Stroke In Vitro

**FIGURE 5:**
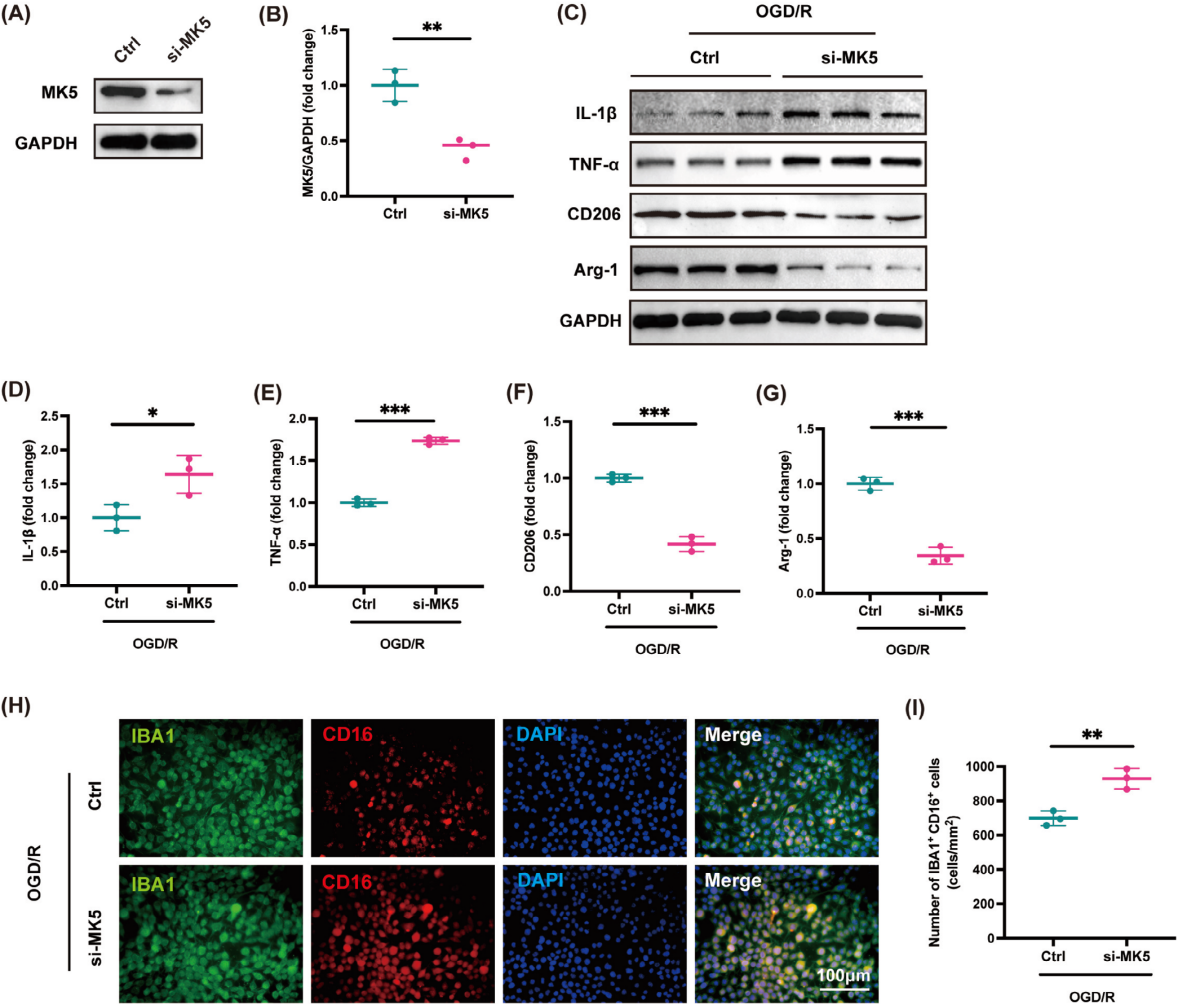
MK5 gene knockout in microglia promotes microglial activation in vitro. (A, B) Effects of MK5 expression in BV2 cells after control (Ctrl) or MK5 siRNA (si‐MK5) transduction, as determined by Western blot. BV2 cells were transduced with MK5 siRNA for 36 h and then the expression levels of MK5 were measured. *n* = 3/group, **p < 0.01. (C–G) Western blot analysis of pro‐inflammatory cytokines IL‐1β, TNF‐α, and anti‐inflammatory markers CD206, Arg‐1 in BV2 cells transfected with Ctrl or si‐MK5 and subjected to OGD/R. *n* = 3/group, *p < 0.05, ***p < 0.001. (H) Immunostaining for co‐localization of IBA1 and CD16 in BV2 cells transfected with Ctrl or si‐MK5 and subjected to OGD/R. Scale bar: 100 μm. (I) Statistical analysis of IBA1+/ CD16+ cells in BV2 cells transfected with Ctrl or si‐MK5 and subjected to OGD/R. *n* = 3/group, **p < 0.01.

To further investigate the regulatory role of MK5 in the inflammatory response of microglia, MK5 mRNA was transiently silenced in the BV2 microglial cell line using siRNA. OGD/R was applied to simulate ischemia/reperfusion injury in vitro. Western blot analysis confirmed that MK5 protein levels were significantly reduced in BV2 cells transfected with si‐MK5 (Figure 5A,B). Moreover, transfection of BV2 cells with si‐MK5 led to an increase in the expression of pro‐inflammatory cytokines IL‐1β and TNF‐α along with a decrease in the anti‐inflammatory factors CD206 and Arg‐1 under OGD/R conditions (Figure 5C–G). Additionally, immunofluorescent staining demonstrated that transfection of BV2 cells with si‐MK5 increased the number of CD16+/Iba1+ cells under OGD/R conditions (Figure 5H,I). Our data demonstrate that knockdown of MK5 exacerbates OGD/R‐induced activation of BV2 microglia and promotes a pro‐inflammatory response in microglia.

### 3.6 MK5 in Microglia May Regulate Neuroinflammatory Responses by Downregulating HSP27 and Mediating the NF‐κB Signaling Pathway

**FIGURE 6:**
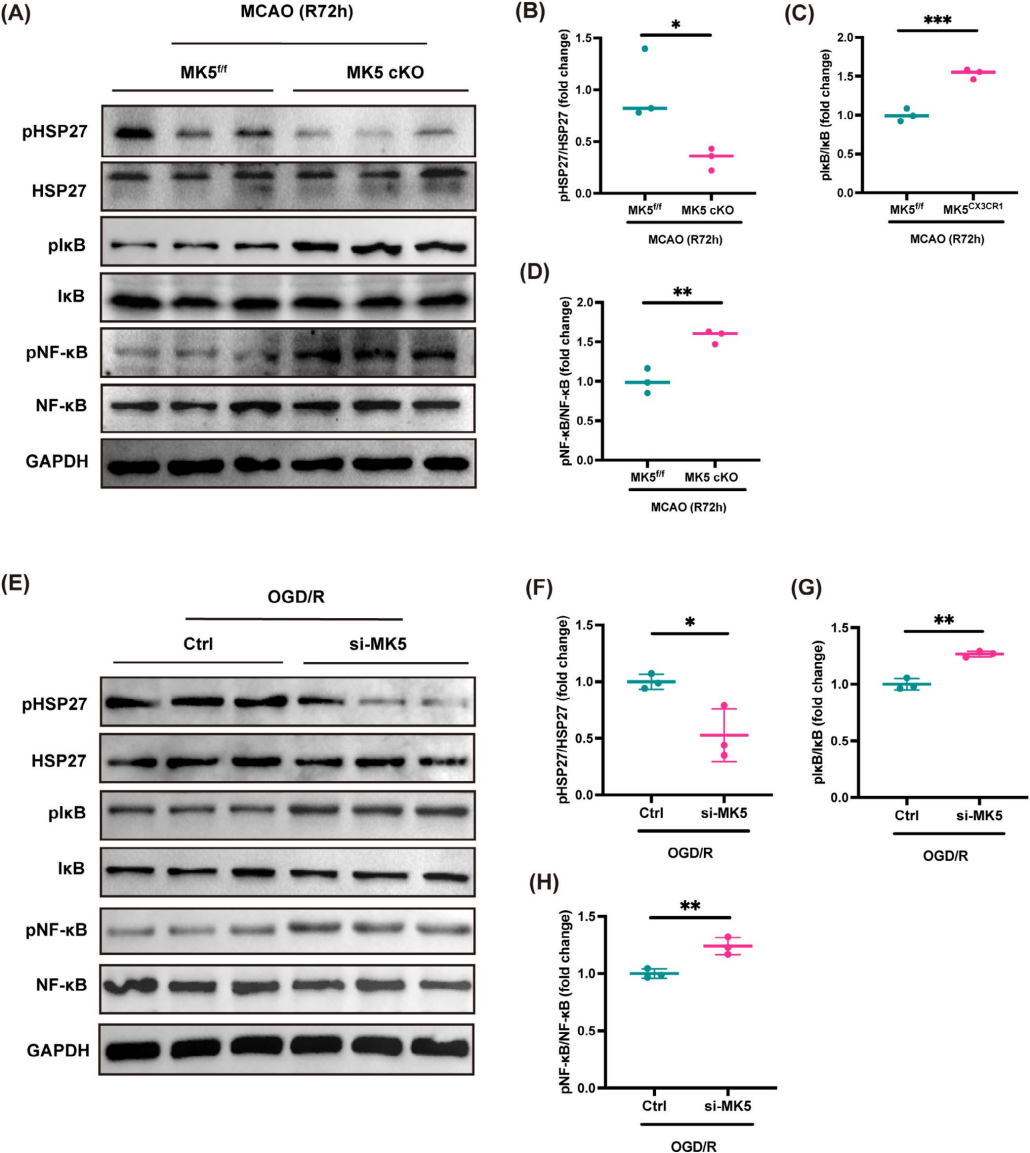
MK5 gene knockout in microglia may participate in neuroinflammatory responses by downregulating HSP27 to mediate the NF‐κB signaling pathway. (A) Western blot analysis of HSP27, pHSP27, IκB, pIκB, NF‐κB, pNF‐κB protein expression in the peri‐infarct cortex of MK5^f/f^ and MK5 cKO mice 3 days post‐MCAO. (B–D) Statistical analysis of the pHSP27/HSP27, pIκB/IκB, and pNF‐κB/NF‐κB ratio. *n* = 3/group, *p < 0.05, **p < 0.01, ***p < 0.001. (E) Western blot analysis of HSP27, pHSP27, IκB, pIκB, NF‐κB, pNF‐κB protein expression in BV2 cells transfected with Ctrl or si‐MK5 and subjected to OGD/R. (F–H) Statistical analysis of the pHSP27/HSP27, pIκB/IκB, and pNF‐κB/NF‐κB ratio. *n* = 3/group, *p < 0.05, **p < 0.01, ***p < 0.001.

Research indicates that MK5 has the ability to trigger the phosphorylation of HSP27, suggesting that HSP27 may be a potential downstream substrate of MK5 [24, 25]. Therefore, we further investigated whether MK5 in microglial cells regulates the NF‐κB signaling pathway via HSP27 following ischemic stroke. Western blot analysis revealed that MK5 knockout in microglia reduced the expression of phosphorylated HSP27 in the ischemic penumbra compared to the control group at 72 h post‐MCAO/R. Additionally, MK5 knockout in microglia was associated with elevated levels of phosphorylated IκB and phosphorylated NF‐κB at 72 h post‐MCAO/R (Figure 6A–D). Furthermore, transfection of BV2 cells with si‐MK5 also led to a decrease in phosphorylated HSP27 expression under OGD/R conditions. In parallel, si‐MK5 transfection resulted in increased levels of phosphorylated IκB and phosphorylated NF‐κB in BV2 cells subjected to OGD/R conditions (Figure 6E–H). These findings confirm that MK5 in microglial cells may participate in neuroinflammatory responses by downregulating p‐HSP27 to mediate the NF‐κB signaling pathway.

## 4 Discussion

MK5, also known as p38‐regulated/activated protein kinase (PRAK), is encoded by Mapkapk5 [26]. Sequence analysis reveals that Mapkapk5 contains between 7 and 18 exons, resulting in multiple splice variants. Although the regulatory mechanisms of MK5 mRNA splicing and the functions of these variants are not fully understood, their temporal and spatial expression suggests involvement in various physiological processes [27]. As a component of the MAPK signaling cascade, MK5 has been implicated in various neurological disorders, including Alzheimer's disease [28], Parkinson's disease [29] and autoimmune encephalomyelitis [30]. However, the role of MK5 in ischemic stroke remains unclear. Our data indicate that genetic knockout of MK5 in microglia significantly impairs neurological motor function in stroke mice and promotes inflammation through microglial activation. These findings suggest that MK5 may have neuroprotective effects, and further research is needed to explore its potential as a clinical therapy for ischemic stroke.

Numerous studies indicate that neuroinflammation plays a crucial role in the pathogenesis and prognosis of ischemic stroke [31, 32]. Microglia, as the primary regulators of neuroinflammatory responses, are crucial for maintaining central nervous system homeostasis and play a pivotal role in both brain development and neurological diseases [33, 34]. Microglia exhibit dual roles in neuroinflammation, which are influenced by their phenotype. Several studies, including our previous findings, suggest that modulating the microglial phenotype from a pro‐inflammatory M1 state to an anti‐inflammatory M2 state may offer a promising therapeutic approach for ischemic stroke [17, 35, 36]. Similar to the observations mentioned earlier, our study found that genetic knockout of MK5 in microglia leads to increased expression of pro‐inflammatory cytokines and decreased expression of anti‐inflammatory cytokines. To our knowledge, this is the first study to demonstrate that MK5 knockout induces a shift in microglia towards a pro‐inflammatory M1 phenotype, revealing a novel role for MK5 in regulating microglial polarization. It is important to note that, consistent with most studies in this field, our research employed a binary M1/M2 polarization classification. However, the notion that microglia are strictly dichotomous and only express M1 or M2 markers has been challenged, as it is considered an oversimplification [37, 38]. Advances in single‐cell technologies and mass cytometry have enabled the discovery of novel microglial subtypes with complex molecular profiles. Consequently, further research is needed to explore the specific roles of MK5 in microglial polarization.

HSP27, a member of the small heat shock protein family, acts as a critical regulatory factor in cellular architecture, cell migration, cell cycle progression, inflammation, gene expression, muscle contraction, signal transduction, differentiation, and apoptosis [39]. Several studies have demonstrated that the activation of HSP27 plays a crucial role in protecting against cerebral ischemia, making it a potential therapeutic target for ischemic conditions [40, 41]. MK5 can be considered an HSP27 kinase that phosphorylates HSP27 in vitro and is involved in distinct pathways [25]. The results of this study also reveal that MK5 may mediate the phosphorylation of HSP27 in the MCAO model. Studies have revealed that the NF‐κB signaling pathway is over‐activated in microglial cells following a stroke, serving as a crucial regulator of the inflammatory response by driving the transcription of downstream pro‐inflammatory cytokines such as IL‐1β, TNF‐α, and IL‐6 [42, 43]. HSP27 may suppress immune inflammation and apoptosis by inhibiting NF‐κB‐dependent pro‐inflammatory signaling pathways in ischemia‐reperfusion injury [44]. This effect may be attributed to its role in preventing the degradation of IκB, an endogenous inhibitor of NF‐κB, as IκB degradation is essential for NF‐κB activation [45]. Our study demonstrated that in both in vivo and in vitro models of ischemic stroke, the genetic knockout of MK5 in microglial cells inhibited HSP27 phosphorylation, increased IκB phosphorylation, enhanced IκB degradation, activated the NF‐κB signaling pathway, and promoted inflammation. These findings indicate that HSP27 serves as a critical mediator of post‐stroke inflammation and suggest a significant role for HSP27 in MK5‐mediated modulation of the NF‐κB signaling pathway during neuroinflammatory responses. However, the precise mechanisms underlying these effects remain to be elucidated and warrant further investigation.

The present study has several potential limitations. First, although we observed a significant effect of MK5 knockout on microglial polarization, the long‐term consequences of these changes, including neuroplasticity and tissue remodeling, were not evaluated. Future research should focus on post‐stroke recovery studies to investigate the ongoing role of MK5 in microglial activation during the chronic phase and its impact on neurovascular remodeling. Second, while our results demonstrate that MK5 knockout in microglia exacerbates neuroinflammation and neurological deficits following ischemic stroke, the effects of MK5 knockout on other brain cells remain unclear. Further investigation into the role of MK5 in other cell types, including neurons and astrocytes, would provide a more comprehensive understanding of its contribution to neuroinflammation and ischemic brain injury. Finally, the therapeutic potential of targeting MK5 remains speculative at this stage. While our data suggest that MK5 may be a promising target for modulating neuroinflammation in ischemic stroke, our study only includes animal and cell‐based experiments, and clinical research is needed for further validation.

## 5 Conclusion

In summary, our findings indicate that MK5 is involved in brain injury and neuroinflammation following cerebral ischemia. Furthermore, the role of MK5 in mediating neuroinflammation after ischemia may be closely associated with its function in the NF‐κB signaling pathway through HSP27 in microglia. Our results suggest that MK5 may represent a potential therapeutic target for the inflammatory response following ischemic stroke.

## Author Contributions

Xingzhi Wang, Guiyun Cui, and Guangci Xu designed the study. Xingzhi Wang, Wenqi Mao, Li Du, Ye Pang, and Fei Wang performed the experiments. Yangdanyu Li and Li Du provided help with data analyses. Xingzhi Wang drafted the manuscript. Guiyun Cui and Guangci Xu provided advice for this manuscript. All authors read and approved the final manuscript.

## Conflicts of Interest

The authors declare no conflicts of interest.

## Supporting information


Table S1.

## Data Availability

The data that support the findings of this study are available from the corresponding author upon reasonable request.
