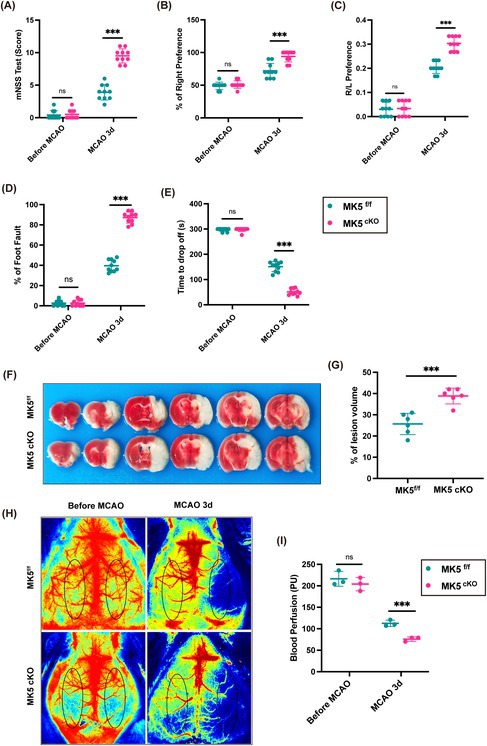# Correction to “MK5 Regulates Microglial Activation and Neuroinflammation in Experimental Stroke Models”

**DOI:** 10.1111/cns.70640

**Published:** 2025-11-02

X. Wang, W. Mao, L. Du, et al., “MK5 Regulates Microglial Activation and Neuroinflammation in Experimental Stroke Models,” CNS Neuroscience & Therapeutics 31, no. 4 (2025): e70395. https://doi.org/10.1111/cns.70395.

In the original version of this article, there was an error in Figure 2H. Specifically, the bottom left image. The correct image is provided below. The correction does not affect the results or conclusions of this paper.

We apologize for this error.