# Supplementary material for: MK5 Regulates Microglial Activation and Neuroinflammation in Experimental Stroke Models
Source: CNS Neurosci Ther. 2025 Apr 16;31(4):e70395. doi: 10.1111/cns.70395 (PMC12001269; doi:10.1111/cns.70395)
Supplement: Supplementary file 1 — Table S1. [file CNS-31-e70395-s001.docx]

Table S1 Primer sequences for qPCR

| **Gene** | **Primers** | **Sequence** |
| --- | --- | --- |
| CD206 | FORWARD | 5’-CCTATGAAAATTGGGCTTACG G -3’ |
|  | REVERSE | 5’-CTGACAAATCCAGTTGTTGAGG-3’ |
| Arg-1 | FORWARD | 5’-CATATCTGCCAA AGACATCGTG-3’ |
|  | REVERSE | 5’-GACATCAAAGCTCAGGTGAATC-3’ |
| TGF-β | FORWARD | 5’-ATCTCGATTTTTACCCTGGTGGT-3’ |
|  | REVERSE | 5’-CTCCCAAGGAAAGGTAGGTGATAGT-3’ |
| TNF-α | FORWARD | 5’-GCTCTTCTGTCTACTGAACTTCGG-3’ |
|  | REVERSE | 5’-ATGATCTGAGTGTGAGGGTCTGG-3’ |
| IL-1β | FORWARD | 5’-AGTTGACGGACCCCAAAAG-3’ |
|  | REVERSE | 5’-TTTGAAGCTGGATGCTCCAT-3’ |
| iNOS | FORWARD | 5’-CTGCAGCACTTGGATCAGGAACCTG3-’ |
|  | REVERSE | 5’-GGGAGTAGCCTGTGTGCACCTGGAA-3’ |
| β-actin | FORWARD | 5’-GGCTGTATTCCCCTCCATCG-3’ |
|  | REVERSE | 5’-CCAGTTGGTAACAATGCCATGT-3’ |
